# Changes in plasma endocan level are related to circulatory but not respiratory failure in critically ill patients with COVID-19

**DOI:** 10.1038/s41598-023-48912-w

**Published:** 2023-12-15

**Authors:** Małgorzata Lipińska-Gediga, Anna Lemańska-Perek, Waldemar Gozdzik, Barbara Adamik

**Affiliations:** 1 Clinical Department of Anesthesiology and Intensive Therapy, Wroclaw Medical University, Borowska 213, 50-556 Wrocław, Poland; 2 Department of Chemistry and Immunochemistry, Wroclaw Medical University, Marii Sklodowskiej-Curie 48/50, 50-369 Wrocław, Poland

**Keywords:** Viral infection, Prognostic markers, Diseases, Infectious diseases

## Abstract

The aim of this prospective, observational study was to assess whether changes in the level of endocan, a marker of endothelial damage, may be an indicator of clinical deterioration and mortality in critically ill COVID-19 patients. Endocan and clinical parameters were evaluated in 40 patients with acute respiratory failure on days 1–5 after admission to the intensive care unit. Endocan levels were not related to the degree of respiratory failure, but to the presence of cardiovascular failure. In patients with cardiovascular failure, the level of endocan increased over the first 5 days (1.63, 2.50, 2.68, 2.77, 3.31 ng/mL, *p* = 0.016), while in patients without failure it decreased (1.51, 1.50, 1.56, 1.42, 1.13 ng/mL, *p* = 0.046). In addition, mortality was more than twice as high in patients with acute cardiovascular failure compared to those without failure (68% vs. 32%, *p* = 0.035). Baseline endocan levels were lower in viral than in bacterial infections (1.57 ng/mL vs. 5.25 ng/mL, *p* < 0.001), with a good discrimination between infections of different etiologies (AUC of 0.914, *p* < 0.001). In conclusion, endocan levels are associated with the occurrence of cardiovascular failure in COVID-19 and depend on the etiology of the infection, with higher values for bacterial than for viral sepsis.

## Introduction

Vascular endothelium creates a “smart connection” between blood and tissue compartments and is involved in haemostasis, vasomotion and vascular permeability, inflammation, and oxidative stress in critically ill patients^1^. In patients with a COVID-19 infection, a relationship between the pathophysiology of respiratory failure and vascular disorders has been demonstrated. SARS-CoV-2 (severe acute respiratory syndrome coronavirus 2) activates systemic inflammation, affects the vascular system and is responsible for the deterioration of patient’s clinical condition from infection to sepsis with subsequent multi-organ failure^2^. Autopsy results of COVID-19 patients showed the presence of endotheliitis and viral inclusions in the endothelial cells and confirmed a higher expression of the ACE2 (Angiotensin-Converting Enzyme 2) receptor on vascular endothelial cells compared to the results from uninfected patients^3^. The ACE2 receptor is a type-I transmembrane glycoprotein that negatively regulates the renin-angiotensin system (RAS) by degrading angiotensin II to the heptapeptide angiotensin 1–7^4^. SARS-CoV-2 infection is a likely cause of endotheliitis in various organs, both as a result of direct involvement of the virus and the host inflammatory response. Moreover, it has been indicated that systemic endothelial cell injury/dysfunction resulting from apoptosis and pyroptosis is the main feature of severe pathophysiology of COVID-19 during the inflammatory phase of the disease^3,5,6^.

The human endocan (endothelial cell specific molecule-1) is a soluble proteoglycan that is constitutively expressed in the endothelial cell network and circulates freely in the bloodstream at a concentration of approximately 1 ng/mL^7,8^). Upregulation of the endocan by proinflammatory cytokines such as tumor necrosis factor alpha, interleukin 1 or microbial lipopolysaccharides reveals its regulatory role in the inflammatory process^9^. Endocan has been shown to bind to LFA-1 (lymphocyte function-associated antigen 1) with high affinity, which inhibits its interaction with the intercellular adhesion molecule-1 (ICAM-1)^7^. It was also confirmed that endocan reduces leukocyte rolling and transmigration, but not firm adhesion^10^. Given that SARS-CoV-2 can cause pneumonia, respiratory injury is expected to be the predominant symptom; however, systemic endotheliitis found post-mortem suggests a significant contribution of endothelial dysfunction to the pathology of COVID-19^11^.

The aim of the study was to investigate whether endocan, as a marker of endothelial injury, indicates the clinical progression of the disease assessed on the basis of clinical scores and the presence of organ dysfunction, and can be useful as a prognostic indicator of mortality in critically ill patients with a SARS-CoV-2 infection. We assessed the course of the disease during the first 5 days of ICU treatment using serial measurements of endocan concentrations and laboratory and clinical parameters in a cohort of COVID-19 patients with severe respiratory failure.

## Materials and methods

### Patients

We prospectively collected samples from patients with a COVID-19 infection admitted to the Department of Anesthesiology and Intensive Therapy of Wroclaw Medical University, Wroclaw, Poland between September 2021 and December 2021. The study protocol was approved by the Bioethical Committee of Wroclaw Medical University (No. 394/2021) and the study was conducted in accordance with the Helsinki Declaration of 1975, as revised in 2008. Informed consent was obtained from the patients or a legally authorized representative.

Inclusion criteria: patients with respiratory failure caused by COVID-19 infection admitted to the ICU (intensive care unit) were enrolled in a consecutive manner; the presence of the SARS-CoV2 virus was confirmed in each patient by a real-time reverse-transcriptase polymerase chain reaction (RT-PCR) assay of nasal or pharyngeal swab probes on admission to the ICU.

Exclusion criteria: a length of ICU stay less than 24 h, a suspicion or confirmation of cancer, treatment with immunosuppressants, ongoing chemotherapy or terminal illness excluded patients from the analysis.

The control group consisted of patients diagnosed with sepsis caused by bacterial pneumonia. Blood samples were collected from these patients once after admission to the intensive care unit. The presence of bacterial pneumonia was confirmed by microbiological diagnosis of bronchial secretion using the rapid PCR Multitest for 20 respiratory pathogens (FilmARRAY respiratory Panel, BioFire Diagnostics, Salt Lake City,UT, USA) and positive cultures of mini-bronchoalveolar lavage (mini-BAL) or bronchoalveolar lavage (BAL) (≥ 10^4^ CFU/mL), and pathological findings in pulmonary X-ray examination. A diagnosis of sepsis was based on the Sepsis-3 definition^12^. Viral infection was an additional exclusion criterion in the control group in addition to the exclusion criteria listed above.

### Data collection

Two clinical scores such as acute physiology and chronic health evaluation (APACHE II)^13^ calculated upon admission to the ICU, and sequential organ failure assessment (SOFA)^14^, calculated daily, were used to assess the clinical status and the extent of multiple organ dysfunction, respectively. All acute organ failures including respiratory, cardiovascular, coagulation system, renal, liver, central nervous system, and endocan level were evaluated in the study group. The results of routine laboratory tests, including leukocytes, platelet count, procalcitonin, c-reactive protein, fibrinogen, d-dimers, and antithrombin III (ATIII), creatinine level and bilirubin level were also included in the analysis. These data were available in the patients’ hospital records. COVID-19 pneumonia was confirmed and characterized by diffuse, bilateral ground-glass opacities on computed tomography scanning. Acute respiratory distress syndrome (ARDS) and the applicable stage were determined according to the Berlin ARDS definition^15^. Endothelial injury was evaluated based on the endocan level^16^. Acute kidney injury (AKI) was defined according to the kidney disease:improving global outcome (KDIGO) definition^17^.

### Sample collection and measurement

Blood samples (2.7 mL) anticoagulated with 3.2% sodium citrate were collected from patients diagnosed with COVID 19 on the day of admission to the ICU and on the 2nd, 3rd, 4th, and 5th day of treatment. Plasma was immediately separated (centrifugation at 2000 × *g* for 10 min), aliquoted, and stored at − 70 °C for further analysis. In the control group, a blood sample (2.7 mL) anticoagulated with 3.2% sodium citrate was collected only once on the day of admission to the ICU. The quantitative determination of the endocan level was performed with an enzyme-linked immunosorbent assay (Cohesion Biosciences, London, UK). All measurements were done in duplicate with appropriate controls. All analyses were performed at the Department of Chemistry and Immunochemistry of Wroclaw Medical University.

### Statistical analysis

Statistical analysis was carried out in the STATISTICA 13.3 package, under license of the Wroclaw Medical University, Wroclaw, Poland. The distribution of variables was not normal based on a Shapiro–Wilk test. Therefore, statistical analysis of the data was performed using nonparametric techniques. The Mann–Whitney U test was used to compare differences between two independent groups. The Friedman repeated-measures ANOVA on ranks was applied to repeated measurements. Continuous variables are presented as medians with 25^th^ and 75^th^ percentiles while categorical variables are summarized as counts and fractions. The relationship between the endocan level and other parameters was assessed with a Spearman’s rank correlation test. Statistical significance was determined as *p* ≤ 0.05.

## Results

### Patient characteristics

**Figure 1:**
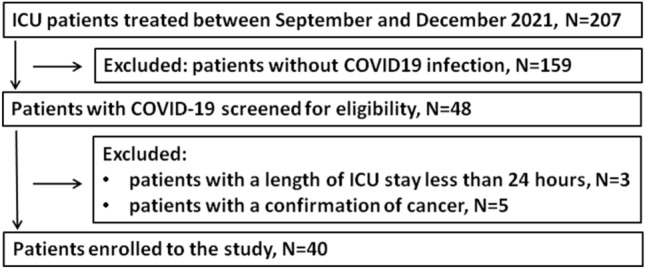
Flow diagram of the study.

A total of 207 consecutive patients who were treated in the ICU between September 2021 and December 2021 were screened for inclusion/exclusion criteria. The presence of SARS-CoV2 was confirmed in 48 patients on admission to the ICU. Out of this number, 8 patients were excluded (3 patients with a length of ICU stay less than 24 h and 5 with a confirmation of cancer). Finally, 40 patients were included in the analysis. The flow diagram of the study is presented in Fig. 1. The median age of the patients was 63 years (IQR 53–71), 63% were male (n = 25). Acute respiratory distress syndrome (ARDS) was confirmed in all patients on admission to the ICU. Sepsis of viral origin, according to the Sepsis-3 definition, was diagnosed in all patients on ICU admission. The ICU mortality was 55%; the baseline characteristics based on the survival status are presented in Table 1. The patients who eventually died were significantly older, and other parameters evaluated at baseline did not differ significantly between survivors and non-survivors.

**Table 1:** Baseline characteristics of the study groups. COVID-19 patients were compared in two subgroups: survivors (N = 18) versus non-survivors (N = 22). In addition, all COVID-19 patients were compared patients diagnosed with bacterial pneumonia.

Parameter	COVID-19	COVID-19	p*	All	All bacterial	p**
	survivors	non-survivors		COVID-19	pneumonia	
	N = 18	N = 22		N = 40	N = 20	
Demographic characteristic						
Age (year)	55	68	0.033	63	65	0.342
	(49–62)	(53–76)		(53–71)	(55–72)	
Male, n (%)	10	15	0.679	25	14	0.565
	(59)	(65)		(63	(70)	
Coexisting conditions, n (%)						
Coronary heart disease	1 (6)	7 (30)	0.061	8 (20)	4 (20)	0.641
Hypertension	6 (33)	3 (14)	0.105	9 (23)	9 (45)	0.073
Diabetes mellitus	3 (17)	2 (9)	0.354	5(13)	3 (15)	0.539
Chronic kidney disease	0	6 (27)	0.026	6 (15)	4 (20)	0.441
Obesity	2 (11)	2 (9)	0.573	4 (10)	0	0.215
COPD	0	1 (5)	0.550	1 (3)	1 (5)	0.544
Asthma	0	1 (5)	0.575	1 (3)	0	0.666
Pregnancy	2 (11)	0	0.174	2 (5)	0	0.441
Inflammation parameters						
Leukocytes [10^3^/uL]	12.02	13.77	0.413	12.96	14.10	0.968
	(9.43–14.90)	(9.69–16.54)		(9.47–16.22)	(5.59–26.77)	
Procalcitonin (ng/mL)	0.16	0.65	0.337	0.41	24.15	< 0.001
	(0.12–0.80)	(0.14–1.76)		(0.13–1.15)	(9.63–88-58)	
C-reactive protein (mg/L)	170	133	0.653	150		
	(87–273)	(72–254)		(76–255)		
Endocan (ng/mL)	1.48	1.57	0.891	1.57	5.25	< 0.001
	(0.95–2.15)	(0.71–2.25)		(0.93–2.25)	(3.79–7.02)	
Clinical scores (points)						
SOFA	9	8	0.806	8	8	0.589
	(7–9)	(7–10)		(7–10)	(7–9)	
APACHE II	13	20	0.054	17	22	0.081
	(11–18)	(15–23)		(12–22)	(15–24)	
ICU LOS (days)	14	13	0.682	14	15	0.387
	(9–30)	(8–18)		(8–24)	(9–35)	

p*-value represents the differences between COVID-19 survivors and non-survivors; p**-value represents the differences between total COVID-19 and patients with bacterial pneumonia. Continuous variables are presented as medians with 25th and 75th percentiles while categorical variables are summarized as counts and fractions.

Additionally, a control group was included to investigate whether there was a difference in endocan concentrations based on the etiology of respiratory infection (viral vs. bacterial etiology). The control group consisted of 20 patients diagnosed with bacterial pneumonia; baseline characteristics of patients with bacterial pneumonia and their comparison with patients with COVID-19 are presented in Table 1.

### Endocan levels in survivors and non-survivors

The endocan level was not an indicator of mortality. Differences in the plasma endocan levels between survivors and non-survivors did not reach statistical significance on any of the observation days (*p* > 0.05). Furthermore, analysis of changes in endocan levels over time (Friedman repeated-measures ANOVA) showed no significant changes in either survivors (1.48, 1.66, 2.12, 1.78, and 1.72 ng/mL on day 1, 2, 3, 4, 5, respectively) or non-survivors (1.57, 2.31, 2.42, 2.72, and 2.78 ng/mL on day 1, 2, 3, 4, 5, respectively). The diagnostic value of baseline endocan as a mortality predictor was statistically not significant (AUC 0.591, 95% CI 0.446–0.736, *p* = 0.218).

### Organ failure and endocan levels in COVID-19 patients

The clinical condition and multi-organ failure of COVID-19 patients was assessed daily using the SOFA score. The median score was similar in survivors and non-survivors at ICU admission and significantly higher in non-survivors on days 2, 3, 4 and 5 (Table 2). Below, the relationship between the endocan level and the occurrence of organ failure (cardiovascular, respiratory, renal, coagulation system, liver, and central nervous system) is analyzed.

**Table 2:** Sequential organ failure assessment scores (SOFA) calculated daily in COVID-19 patients.

	SOFA score				
	Day 1	Day 2	Day 3	Day 4	Day 5
Survivors, N = 18	9 (7–9)	7 (6–9)	7 (6–7)	6 (5–8)	6 (3–8)
Non-survivors, N = 22	8 (7–10)	8 (7–12)	8 (7–11)	9 (7–11)	8 (6–11)
*p*	0.869	0.047	0.039	0.002	0.031

Variables are presented as medians with interquartile range. *p*-value represents the differences between survivors and non-survivors.

#### Acute cardiovascular failure and endocan levels

**Figure 2:**
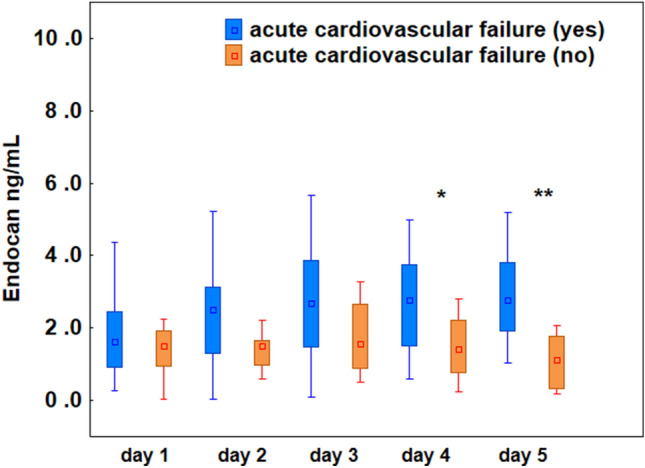
Plasma levels of endocan measured in patients with COVID-19 on the 1st, 2nd, 3rd, 4th, and 5th day of the study. The difference between patients with and without cardiovascular failure was significant on the 4th and 5th days of observation (*p ≤ 0.05; ** *p* < 0.005). The box plots represent the median values (midpoint) with interquartile range between the 25th and 75th percentiles (box); the whiskers represent the minimum and maximum values.

Acute cardiovascular failure on admission to the ICU was diagnosed in 27 patients (68%) and 13 patients (32%) had no failure. Among patients with acute cardiovascular failure, 11 required doses of norepinephrine > 0.1 µg/kg/min (cardiovascular SOFA score 4 pts.) and 16 required a norepinephrine dose ≤ 0.1 µg/kg/min (cardiovascular SOFA score 3 pts.). The kinetics of changes in the endocan concentration was different in patients with and without cardiovascular failure. In patients with cardiovascular failure, endocan levels increased over the first 5 days (1.63, 2.50, 2.68, 2.77, 3.31 ng/mL on day 1, 2, 3, 4, 5, respectively, *p* = 0.016), while in patients without cardiovascular failure it decreased (1.51, 1.50, 1.56, 1.42, 1.13 ng/mL on day 1, 2, 3, 4, 5, respectively, *p* = 0.046). In addition to significantly higher endocan levels in patients with acute cardiovascular failure, mortality in this group was twice as high as in patients without failure (68% vs. 32%, *p* = 0.035). Figure 2 illustrates a comparison of endocan concentrations in two patient subgroups depending on the presence of cardiovascular failure; statistically significant differences between the groups were found on the 4th and 5th day of observation.

#### Acute respiratory failure and endocan levels

**Figure 3:**
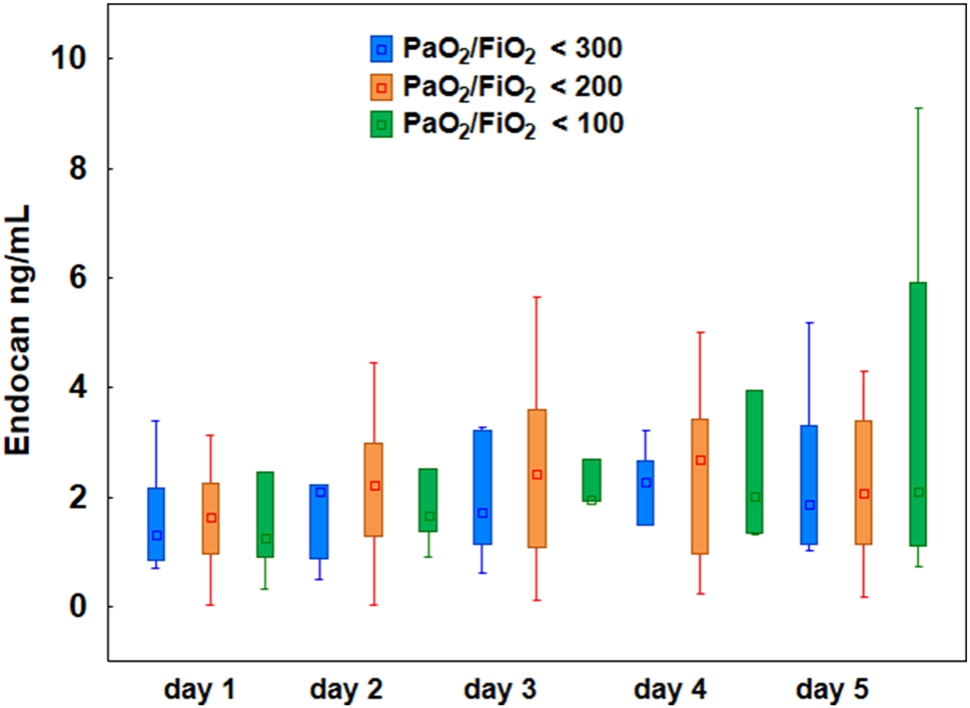
Plasma levels of endocan measured in 40 patients with COVID-19 on the 1st, 2nd, 3rd, 4th, and 5th day of the study. There were no statistically significant differences between groups with PaO_2_/FiO_2_ < 100 mmHg, with 100–199 mm Hg, and from 200 to 299 on any days of observation (Kruskal–Wallis test, *p* > 0.05). The box plots represent the median values (midpoint) with interquartile range between the 25th and 75th percentiles (box); the whiskers represent the minimum and maximum values.

All COVID-19 patients were diagnosed ARDS. To support lung function, mechanical ventilation was applied in all patients (n = 40) and 27% (n = 11) were treated with veno-venous ECMO. On admission to the ICU, the most severe respiratory failure with the lowest PaO_2_/FiO_2_ ratio (< 100) was recorded in 18% of patients (respiratory SOFA score of 4 pts.), PaO_2_/FiO_2_ within the range 100–199 in 67%, (respiratory SOFA score of 3 pts.), and within the range 200–299 in 15% (respiratory SOFA score of 2 pts.). The results of the Kruskal–Wallis test indicated no differences in endocan levels between groups with PaO_2_/FiO_2_ < 100, from 100 to 199, and from 200 to 299 on any of the observation days (*p* > 0.05) (Fig. 3).

#### Acute kidney failure and endocan levels

**Figure 4:**
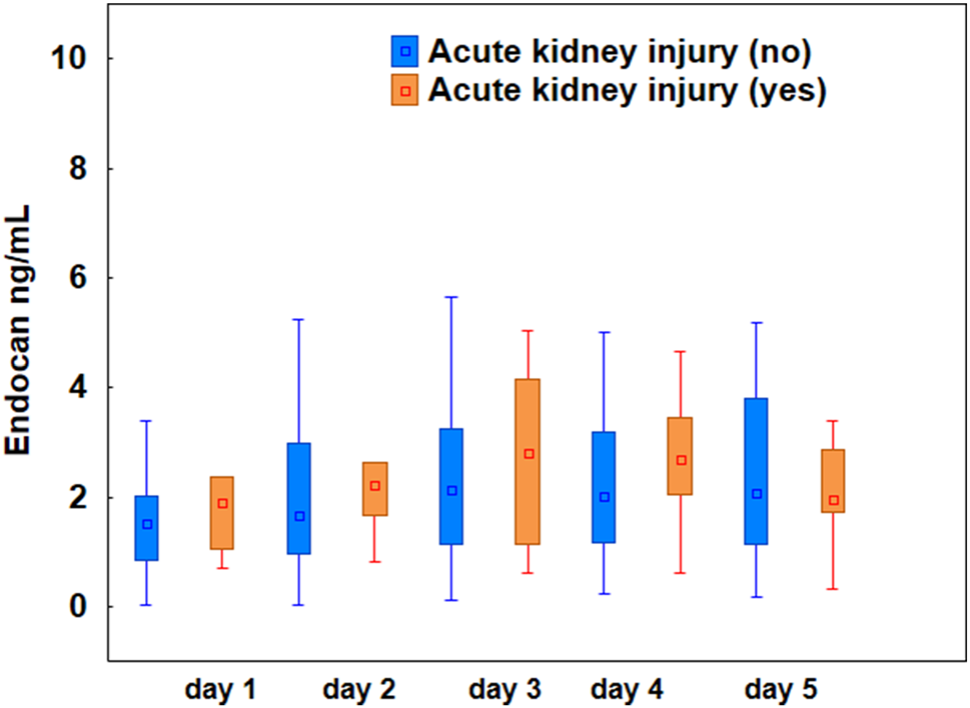
Plasma levels of endocan measured in patients with COVID-19 on the 1st, 2nd, 3rd, 4th, and 5th day of the study. There were no statistically significant differences between patients with and without acute kidney injury on any observation day (Mann–Whitney test, *p* > 0.05). The box plots represent the median values (midpoint) with interquartile range between the 25th and 75th percentiles (box); the whiskers represent the minimum and maximum values.

On admission to the ICU, 22 patients (55%) were diagnosed with an acute kidney injury (AKI) and 10 of them (45%) required renal replacement therapy (RRT). There were no differences in endocan levels between groups with and without RRT on any of the observation days (Fig. 4, *p* > 0.05).

#### Coagulation abnormalities and endocan levels

Coagulation parameters were calculated daily (Table 3). Coagulation abnormalities were common in COVID-19 patients. The level of d-dimers was elevated in 98% of patients on ICU admission and the median d-dimers were significantly higher in the non-survivors than in survivors, with *p* = 0.020. Analysis of changes in d-dimer levels over time in each study group using Friedman repeated-measures ANOVA showed significant changes in non-survivors, but this result was not confirmed in the post- hoc analyses (*p* > 0.05). The level of fibrinogen was elevated in 78% of patients on ICU admission and the median fibrinogen was similar in the non-survivors and survivors. Platelet counts were normal in a majority of patients (85%) and low platelets (< 150 × 10^9^/L) were recorded in 6 patients on ICU admission. Analysis of changes in platelet counts over time in each study group using Friedman repeated-measures ANOVA showed significant changes in non-survivors, but this result was not confirmed in the post- hoc analyses (*p* > 0.05). In addition, there were no significant differences in platelet counts between the survivors and non-survivors. Similarly, the ATIII level was normal in a majority of patients (88%) and low ATIII on ICU admission (< 80%) was recorded in 5 patients. There were no significant differences in ATIII between the survivors and non-survivors. In addition, the Spearman rank correlation test was used to assess the relationship between the endocan level and coagulation parameters. There was no significant correlation between endocan levels and any of the coagulation parameters at any time point during the five-day observation period, except for a significant negative correlation between endocan and fibrinogen in the group of survivors on day 5 (R -0.721, *p* = 0.018).

**Table 3:** Coagulation parameters calculated daily.

	Day 1	Day 2	Day 3	Day 4	Day 5	p*
D-dimers, normal range < 0.5 mg/L						
Survivors	1.3 (1.0–2.2) **	1.5 (1.0–4.4)	1.6 (1.0–7.1)	1.7 (1.3–21.3)	1.8 (1.3–12.6)	0.346
Non-survivors	6.9 (1.5–20.6)	5.4 (1.5–7.4)	5.1 (1.5–5.5)	1.9 (1.1–5.1)	3.3 (1.2–14.4)	0.022
Fibrinogen, normal range 2.00—4.00 g/L						
Survivors	5.4 (4.3–7.5)	5.4 (5.5–5.5)	6.1 (5.1–6.8)	5.7 (5.2–6.7)	5.5 (4.5–8.7)	0.943
Non-survivors	5.2 (3.5–7.8)	4.5 (3.6–6.3)	4.9 (3.9–5.8)	4.5 (3.5–5.8)	4.61 (3.4–7.1)	0.907
Platelets, normal range > 150 × 10^9^/L						
Survivors	244 (179–405)	213 (166–287)	215 (134–295)	180 (127–313)	191 (148–329)	0.568
Non-survivors	210 (182–295)	212 (159–309)	233 (165–325)	209 (175–281)	198 (125–234)	0.027
ATIII, normal range > 80%						
Survivors	107 (83–127)	89 (86–111)	100 (98–108)	95 (64–113)	99 (65–111)	0.067
Non-survivors	91 (83–99)	94 (88–120)	97 (89–119)	95 (90–107)	110 (107–119)	0.292

*Represents changes over time in each study group (Friedman repeated-measures ANOVA test);

**Represents the statistically significant difference between survivors and non-survivors (*p* ≤ 0.05 Mann–Whitney U test).

#### Liver failure and endocan levels

Bilirubin level was normal in 77% (N = 31) of COVID-19 patients and elevated in 23% (N = 9: bilirubin level between 1.2 and 1.9 mg/dL in 4 patients and between 2.0—5.9 mg/dL in 5 patient. Differences in the plasma endocan levels between patients with the normal and elevated bilirubin level did not reach statistical significance on any of the observation days (*p* > 0.05). Furthermore, analysis of changes in endocan levels over time (Friedman repeated-measures ANOVA) did not show significant changes in patients with normal bilirubin levels (1.51, 1.67, 2.02, 1.94, and 1.85 ng/mL on day 1, 2, 3, 4, 5, respectively, *p* = 0.571) or in patients with elevated bilirubin levels (1.63, 2.50, 2.68, 3.21, and 2.05 ng/mL on day 1, 2, 3, 4, 5, respectively, *p* = 0.751).

#### Glasgow coma scale

On ICU admission*,* GCS was 15 or between 13–14 pts. in the majority of patients (N = 36) and decreased in 4 patients (in 1 patient between 10 and 12 pts. and in 3 between 6 and 9 pts.). Baseline endocan concentration was 1.51 ng/mL (IQR 0.87–2.01) in patients with with GCS 15 or between 13–14 pts. and 1.77 ng/mL (IQR 1.48–3.55) in patients with GCS < 13 points, *p* = 0.343.

### Endocan as a marker differentiating COVID-19 from bacterial infection

**Figure 5:**
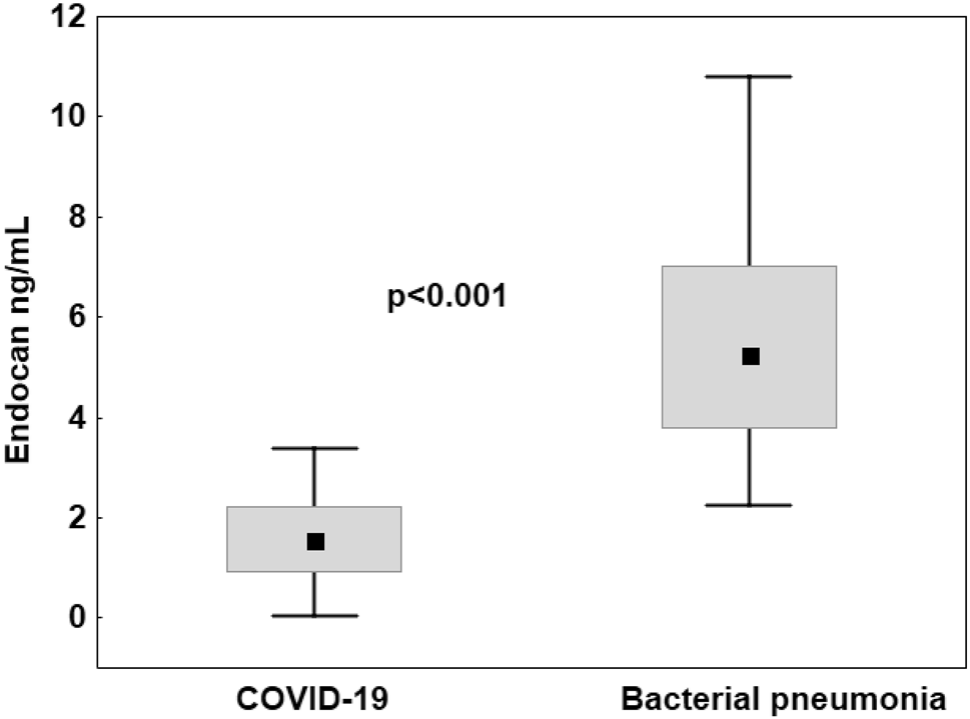
Plasma levels of endocan measured in patients with COVID-19 and in patients with bacterial pneumonia on admission to the intensive care unit. The box plots represent the median values (midpoint) with interquartile range between the 25th and 75th percentiles (box); the whiskers represent the minimum and maximum values.

To assess whether there was a difference in endocan concentrations based on the etiology of the infection, we compared endocan levels in viral (COVID-19) and bacterial (pneumonia) respiratory infections. Therefore, in addition to blood samples from COVID-19 patients, samples were also collected from 20 patients with bacterial pneumonia (control group) on the day of ICU admission. A comparison of all COVID-19 patients and all bacterial pneumonia patients is shown in Table 1. The group of COVID-19 patients and the control group were similar in age (*p* = 0.342), gender (*p* = 0.565), APACHE II score (*p* = 0.081), and SOFA score (*p* = 0.589). Patients in the control group had significantly higher PCT level than COVID-19 patients (24.15 vs. 0.41 ng/mL, *p* < 0.001). As shown in Fig. 5, baseline endocan levels were significantly lower in COVID-19 compared to the control group (1.57 ng/mL, IQR: 0.93—2.25 vs. 5.25 ng/mL, IQR: 3.79–7.02; *p* < 0.001).

**Figure 6:**
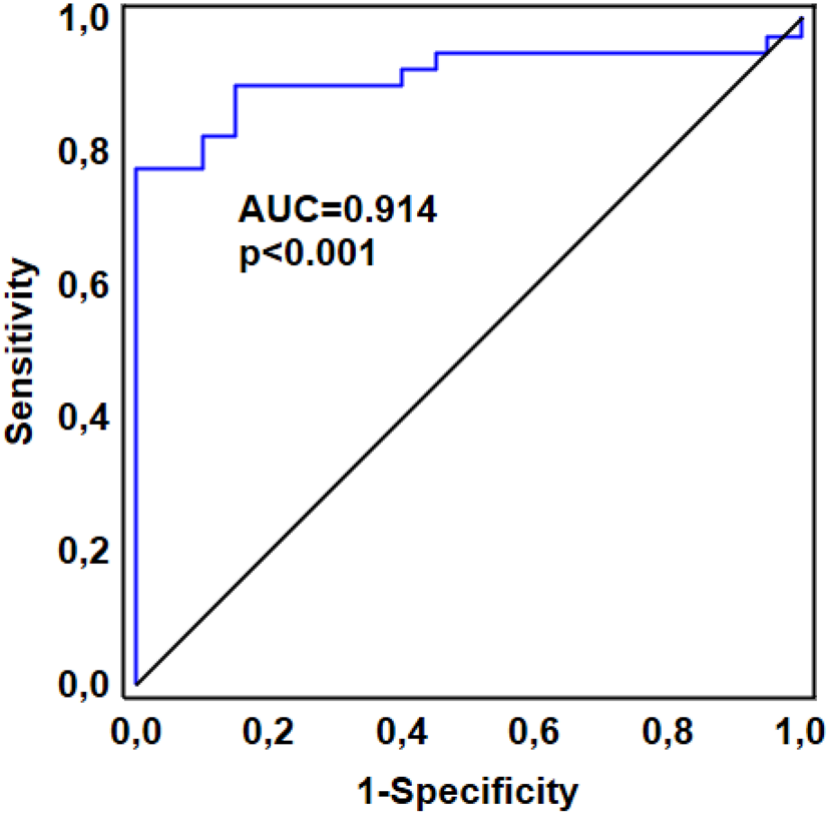
Receiver operating characteristic curve for baseline endocan levels for predicting a COVID-19 infection: the area under the curve (AUC) 0.914 (95% CI 0.838–0.989, *p* < 0.001), best cutoff value 3.39 ng/mL; sensitivity 0.90, specificity 0.85; positive predictive value 0.92, negative predictive value 0.81.

In the ROC (Receiver Operating Characteristic) curve analysis, the baseline endocan levels showed very good ability to discriminate between a viral and bacterial infection with the AUC of 0.914 (95% CI 0.838–0.989, *p* < 0.001). The optimal threshold value was 3.39 ng/mL (sensitivity 90%, specificity 85%) (Fig. 6).

## Discussion

This study assessed the involvement of the endothelium in the pathophysiology of a SARS-CoV-2 infection based on changes in plasma endocan levels in patients diagnosed with COVID-19; patients with critical COVID-19 with the most severe symptoms requiring ICU treatment were included. All patients were diagnosed with acute respiratory failure and all needed mechanical ventilation to support lung function on admission to the ICU. We found that endocan levels were not related to the degree of respiratory failure, but to the presence of cardiovascular failure. In patients with cardiovascular failure, the level of endocan increased on subsequent days, reaching the highest value on the last day of observation, while in the group without failure it decreased. In addition, mortality was more than twice as high in patients with acute cardiovascular failure compared to those without failure. We also found that endocan levels were different for viral and bacterial infections in the study cohort, with significantly lower levels of endocan in COVID-19 patients than in patients with bacterial pneumonia on ICU admission.

SARS-CoV-2 binds to ACE2 on the cell membrane of the host cells. ACE2 has been found in arterial and venous endothelia cells in various human tissues^4^. The functional unit of the lung is the alveolar-capillary interface, and the pulmonary capillary network is the largest vascular bed in the body^18^. A SARS-CoV-2 infection induces viral pneumonia, which leads to acute respiratory failure in up to 20% of symptomatic patients, with ARDS being the most severe form of lung injury^11^. COVID-19-related ARDS is considered atypical, with the main characteristic being a discrepancy between hypoxaemia severity and relatively good respiratory mechanics. Symptoms are linked to endothelial damage and ventilation–perfusion mismatch, which are mainly responsible for the severity of hypoxaemia and promoting immunothrombosis^19^. Taking these facts into account, the term MicroCLOTS (microvascular COVID-19 lung vessels obstructive thromboinflammatory syndrome) was proposed for classification of COVID-19—related ARDS^20^.

Endocan is expressed in lung endothelial cells and upregulated during a systemic inflammatory response^21^. In the cohort assessed in our study (critically ill COVID-19 patients), endocan was not a predictor of mortality and no significant differences in endocan levels were found between survivors and non-survivors. Similar results were demonstrated by Guzel et al.^22^ in the population of COVID-19 patients hospitalized for mild/moderate pneumonia and severe pneumonia. They showed that serum endocan levels were not associated with the degree of pneumonia or 28-day survival^22^. These results contrast with those of Medetalibeyoglu et al.^23^ who found that in a general population of hospitalized COVID-19 patients, the serum endocan level had a sensitivity of 97% and a specificity of 85% as a predictor of poor prognosis defined as ICU admission and mortality. In another study, the endocan level measured at admission also predicted a poor prognosis for COVID-19, with a sensitivity of 86.7% and a specificity of 50%^24^. In our study, in the population of ICU patients with COVID-19, the prognostic value of the endocan level as a predictor of mortality was not significant therefore, no cut-off point was established.

The results of our study showed that there was no significant difference in endocan concentration in patients with different degrees of severity of COVID-19—related respiratory failure assessed by the oxygenation index. This observation is consistent with the previously published work of Orbegozo et al.^25^, who showed no correlation between endocan level and oxygenation index in patients with ARDS; however, plasma endocan concentrations measured early in the course of ARDS predicted longer (more than 10 days) duration of mechanical ventilation. Similar results were shown by Pascreau et al.^21^ indicating no difference in the endocan level between COVID-19 patients who developed ARDS and those who did not. In a commentary on the study by Pascreau, Honore et al.^26^ stated that in SARS-CoV-2-associated pneumonia/ARDS, the increase in endocan levels was caused by high endothelial activity and thrombotic complications rather than by diffuse alveolar damage. These observations are consistent with the results of postmortem lung biopsies, which revealed endothelial and vascular injury as a predominant pathology in the course of COVID-19^27^. Moreover, Leisman et al.^28^ pointed out that the shift from alveolar to endothelial injury indicates that the course of COVID-19 involves a transition from a pathology located primarily in the lungs to a pathology of the systemic circulation with endothelial activation/injury.

Hemostatic disorders in critically ill COVID-19 patients are caused by hypoxia combined with inflammation and thrombotic manifestations, endotheliopathy-associated vascular microthrombotic disease (EA-VMTD) is the clinical presentation of these changes^29,30^. In our study, coagulation abnormalities were common in COVID-19 patients. Fibrinogen and d-dimer levels were elevated in the majority of patients on ICU admission, indicating thrombotic complications, with d-dimer levels being significantly higher in non-survivors than in survivors. In earlier research by Helms et al.^31^, more than 95% of COVID-19 patients had elevated d-dimers and fibrinogen levels at baseline, but platelet counts and ATIII levels remained within normal ranges in 80%, and 66% of patients respectively. In viral sepsis, toxins such as the S protein of the SARS-CoV-2 virus activate the complementary system, leading to the formation of C5b-9-MAC (membrane attack complex), which neutralizes and kills the pathogen and infected host cells. MAC may cause morphological (membrane pore formation) and functional changes of endothelial cells, initiating disseminated endotheliopathy, if endothelial protecting CD59 membrane glycoprotein is underexpressed^32^. In COVID-19 patients, postmortem analysis of multiple organs revealed perivascular immune cell infiltrates, endothelial apoptosis, and decreased endothelial barrier properties as visualized by fibrinogen leakage^33^. In our study, fibrinogen levels were lower in the group of non-survivors than in survivors throughout the observation period with a strong negative correlation between fibrinogen and endocan at the end of observation. This may suggest that fibrinogen leakage was more intense and irreversible, with lower fibrinogen levels measured in the blood in the group of non-survivors.

The Cox et al.^34^ demonstrated a significant correlation between endocan and endothelium-dependent vascular dysfunction in a systemic inflammation induced by experimental endotoxemia. In our study the endocan level starting from the 4th study day was significantly higher in patients presenting with cardiovascular failure in comparison to those without failure. In addition to significantly higher endocan levels in patients with cardiovascular failure, mortality in this group was twice as high as in patients without failure. A similar observation applies to changes in the SOFA score in both the survivor and non-survivor groups: there was a continuous decrease in the SOFA score and endocan level in survivors and an increase in non-survivors, with a significant difference in the SOFA score starting from the 2nd day of the study. In our opinion, all these results indicate that in COVID-19 critically ill patients with multiorgan dysfunction syndrome, the circulatory failure and its progression is the main factor determining mortality. Corroborating our results, Kim^35^ previously showed that endocan was elevated in COVID-19 and furthermore, a decrease in the levels of endothelial injury—related biomarkers was associated with clinical improvement. In another study, Leisman et al.^28^ showed that elevated levels of endocan and other markers of endothelial injury coincided with increased extrapulmonary organ dysfunction and were more strongly associated with 28-day outcome than alveolar markers. These findings are consistent with those of Stahl et al.^36^ who found that endothelial glycocalyx shedding could predict widespread endothelial injury in severe COVID-19.

In our study, the baseline endocan level was significantly lower in COVID-19 compared to ICU patients with bacterial pneumonia. In the studied cohort of ICU patients, the baseline endocan level showed very good ability to discriminate between a viral and bacterial infection with the optimal threshold value of 3.39 ng/mL. In viral infections, interferon gamma inhibits tumor necrosis factor alpha—induced upregulation of endocan expression and these results likely reflect pathophysiological differences in endothelial injury in severe viral infections compared with severe bacterial infections^9^.

In this study there are some limitations which might affect our results. It is a single center study with a rather small sample size and our observations require confirmation on a larger cohort.

## Conclusions

Based on our results, it may be concluded that in critically ill COVID-19 patients, changes in plasma endocan concentration are not related to the severity of respiratory dysfunction but to the presence of circulatory failure. This may suggest the need to revise the understanding of the role of endocan as a biomarker related more to a circulatory failure and to a lesser extent to respiratory failure. Equally important seems to be the observation that changes in plasma endocan concentrations depend on the etiology of the infection, suggesting that the vasculopathy associated with COVID-19 appears to be significantly different from that occurring in bacterial infections.

## Data Availability

The datasets analyzed during the current study are available from the corresponding author on reasonable request.
